# BCC-NER: bidirectional, contextual clues named entity tagger for gene/protein mention recognition

**DOI:** 10.1186/s13637-017-0060-6

**Published:** 2017-05-05

**Authors:** Gurusamy Murugesan, Sabenabanu Abdulkadhar, Balu Bhasuran, Jeyakumar Natarajan

**Affiliations:** 1 Data Mining and Text Mining Lab, Department of Bioinformatics, Bharathiar University, Coimbatore, Tamilnadu 641046 India; 2 Center for Computational Biology, DRDO-BU Center for Life Sciences, Bharathiar University, Coimbatore, Tamilnadu 641046 India

**Keywords:** Biomedical text mining, Named entity recognition, Conditional random fields, Hybrid NER approaches, Margin-infused relaxed algorithm, Bidirectional parsing

## Abstract

Tagging biomedical entities such as gene, protein, cell, and cell-line is the first step and an important pre-requisite in biomedical literature mining. In this paper, we describe our hybrid named entity tagging approach namely BCC-NER (bidirectional, contextual clues named entity tagger for gene/protein mention recognition). BCC-NER is deployed with three modules. The first module is for text processing which includes basic NLP pre-processing, feature extraction, and feature selection. The second module is for training and model building with bidirectional conditional random fields (CRF) to parse the text in both directions (forward and backward) and integrate the backward and forward trained models using margin-infused relaxed algorithm (MIRA). The third and final module is for post-processing to achieve a better performance, which includes surrounding text features, parenthesis mismatching, and two-tier abbreviation algorithm. The evaluation results on BioCreative II GM test corpus of BCC-NER achieve a precision of 89.95, recall of 84.15 and overall F-score of 86.95, which is higher than the other currently available open source taggers.

## Introduction

Scientific literature is the major source of biomedical knowledge, and the interest in developing automated text mining solutions to extract useful information from biomedical text is increasing every year. Bio-named entity recognition (NER) is the key step for such information extraction from biomedical literature [1–5].

Biomedical-named entities include genes, proteins, RNA, cell, and cell-line. NER in the biomedical domain is generally considered to be more difficult than other domains such as newswire as there is no standard nomenclature naming biomedical entities like genes and protein names resulting in ambiguity, and further, there are millions of biomedical entity names in use and more entities are added regularly [2, 3]. Moreover biomedical entities such as gene and protein names have similar morphology and context [6].

The commonly used techniques for NER task are rule-based approaches [4], dictionary-based approaches [3], machine learning approaches [2], and recent hybrid systems which use a combination of two or more approaches. Presently, hybrid approaches give best results in NER task [4, 5]. To understand the current state-of-the-art, we briefly introduce some of the recent hybrid approaches explored for biomedical NER task. Raja et al. [4] used a hybrid named entity rule-based tagger with 14 hand-crafted rules and a set of post-processing methods and an abbreviation algorithm to tag the human gene/proteins from biomedical articles.

Leaman et al. [2] proposed a machine learning-based open source biomedical named entity system which was a combination of conditional random fields (CRF) and some post-processing methods to tag gene/proteins. Campos et al. [3] designed a biomedical hybrid tagger with machine learning algorithm and lexicon-based approaches. Zhu et al. [5] used both support vector machines (SVM) and CRF for better performance. SVM, a binary classifier, was used to separate the biological terms from non-biological terms, and CRF was used to determine the types of biological terms. Thus, the results of SVM as well as CRF were fused and a useful algorithm was developed after applying two rules.

**Fig. 1:**
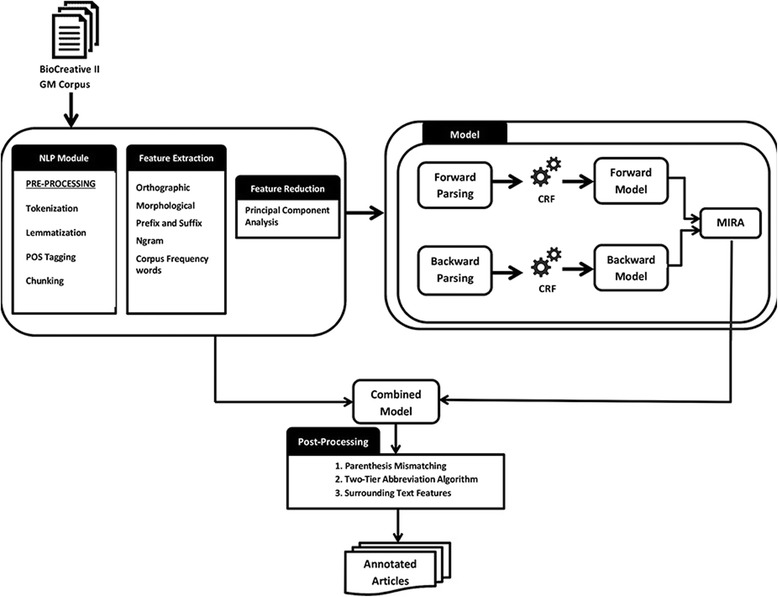
The workflow of various modules in BCC-NER

In this paper, we describe our hybrid approach, namely BCC-NER (bidirectional, contextual clues named entity tagger for gene/protein mention recognition) which uses bidirectional parsing with the tagging results of forward and backward models combined and trained using CRF model. In order to achieve a better performance, we further applied surrounding text features, parenthesis mismatching, and two-tier abbreviation algorithms post-processing steps. Figure 1 depicts the workflow of various modules in BCC-NER.

## Materials and methods

BCC-NER is the hybrid named entity recognition system trained and tested on BioCreative II GM corpus [7]. The system is composed of the following three modules.

1. Text processing module which includes NLP preprocessing, feature extraction, and selection
2. CRF training module, which uses bidirectional CRF for learning and labeling in both directions and model integration using MIRA
3. Post-processing module, which includes contextual clues and abbreviation identification algorithm.

The details of each module are described in the following sections.

### Text processing

#### Text preprocessing

In order to prepare the corpus for feature extraction and NER, the following preprocessing steps were applied initially: (i) sentence splitting for splitting the articles or abstracts to sentences, (ii) tokenization for splitting the sentences into individual tokens, (iii) lemmatization to convert the tokens to the basic form of the word, (iv) POS tagging, and (v) chunking. OpenNLP [8] was used for sentence splitting, tokenization, POS tagging, and chunking. BioLemmatizer [9] was employed for lemmatization.

#### Feature extraction

After preprocessing, for each token, various kinds of features were extracted. These features were based on the local context of each token such as orthographic, morphological, prefix, and suffix features. Orthographic features include the rules of spelling, capitalization, digitization, and punctuation. Morphologic features include identification of the role of words in sentences and their prefixes/suffixes as features. In addition to those above, the features of the preceding two words and the next two words are also used as offset conjunction features. In total, we have used 32 features which are illustrated in Table 1.

**Table 1:** Examples of orthographic, morphologic, and prefix-suffix features

Feature	Example	Feature	Example
INITCAPS	Albumin	HAS_QUOTE	gstC’ mutans
ALLCAPS	SGPT	HAS_SLASH	P42/44
ENDCAPS	IgA	END_PLUS	HexA+
UPPER-LOWER	Serum ACTH	END_QUOTE	C’
TWOCAPS	LH	HASDASH	Ap-2
THREECAPS	HMG	INITDASH	-beta
MORECAPS	GGTP	ENDDASH	CD45-
MIXEDCAPS	EcoRI	2PREFIX	Fi(fibrin)
LOWERCASE	Calcitonin	3PREFIX	Fib(fibrin)
ENDDIGIT	cna1	4PREFIX	Fibr(fibrin)
ALPHANUMERIC	p53	2SUFFIX	in(fibrin)
SINGLECHAR	R	3SUFFIX	rin(fibrin)
NUMBERS_LETTERS	UR2	4SUFFIX	brin(fibrin)
HASDIGIT	E6	HASGREEK	TNF-alpha
GREEK	Alpha	HASROMAN	factor II
ROMAN	I,II,IV	PUNCTUATION	(,).,

In addition to the above basic features, we have also used the general NER features such as N-gram [10] and corpus frequency words [11] to improve the performance of the tagger. For the N-gram feature, we used character N-grams using a sliding window of size 4. The sliding window starts from the beginning to four characters of each token [10].

In our initial tagging, we found some of the tokens tagged as a bio-entity in one sentence but not tagged in some other sentence due to the variation in sentence structure. For example, the gene *COR* was tagged as gene in one occurrence but not in the second in the same abstract (BC2GM000136143). In order to overcome this issue and uniformly tag such tokens, we used corpus frequency words feature only for noun tokens.

For corpus frequency words, we calculated the total number of times each word or sequences of words occur in the corpus. We took the words or sequences of words with a minimum threshold frequency in the range of (1–10) into consideration.

Notation: We write *θ*freq as the count for set of gene/protein names occurring in the corpus.

1 $$ C f(w)\le\ \theta f req $$

where *cf*(*w*) denotes the *corpus frequency* of the word *w*, i.e., the total number of times *w* occurs in the corpus. In our experiments, we set *θ*freq = 10.

#### Feature selection

Due to the inclusion of rich set of features including word N-grams, the number of features associated with each token is very large and many of them may not be related to the tokens. Further, redundancy may also occur during the training phase of the samples which can cause performance degradation of the tagger. In order to use only the most informative features for classification task and to discard unrelated features, we employed a principle component analysis (PCA)-based feature selection method [12]. PCA is the most commonly used dimension reduction method. The vital scheme of PCA is to shrink the dimensionality of a feature set albeit trying to retain the variance present in the original predictor features to the greatest degree possible [13].

PCA converts the data to a new dimensional space in such a way that the features with the highest eigenvalue component comes to the first coordinate, next, the eigenvalue component on the second coordinate, and so on. The dimensionality of the data is then shrunk by ignoring the lower eigenvalue components. Thus, PCA provides the most essential directions that can efficiently represent the data which is shortly explained below [14].

The full principal component decomposition of data matrix **X** can be given as T = XW where **W** is a 2D matrix whose columns are the eigenvectors of **X** ^T^ **X.** The transformation T = XW maps a data vector x(i) from an original space of *p* variables to a new space of *p* variables which are uncorrelated over the dataset. However, not all the principal components are kept during the transformation. Only the first L principal components produced by using the first L-loading vectors that are kept gives the truncated transformation T_L_ = XW_L_ where the matrix T_L_ now has n rows but only L columns. In other words, PCA learns a linear transformation where the columns of p × L matrix W form an orthogonal basis for the L features that are de-correlated [15]. Among all the data matrices thus transformed to only L columns, this score matrix maximizes the variance in the original data that has been preserved, while minimizing the total squared reconstruction error.

### Bidirectional CRF model and integration

#### CRF

Conditional random fields or CRFs are probabilistic frameworks first introduced by Lafferty et al. for labeling and segmenting sequential data. CRFs are a class of statistical modeling methods often applied in pattern recognition and machine learning problems. Whereas an ordinary classifier predicts a label for a single sample with no regards to “neighboring” samples, a CRF can take the context into account [16]. The CRF learning [17] is given by

2 $$ P\left( A\Big| B\right)=\frac{1}{Z(B)}{\displaystyle {\prod}_{k=0}^n{\varPsi}_k\left( B, A\right)} $$

However, in named entity recognition task, CRF computes the probability P (A|B) of given input sequence B = b_1_….b_n_ in the form of a tokenized text and predictable label sequence A = a_1_…..a_n_ in the form of a labeled tokenized text. In the context of NER, a labeled text indicates I-inside [Gene/Protein], O-outside, and B-beginning [Gene/Protein]. In order to learn conditional distributions between A_k_ and feature functions from the observable data in NER, it is necessary to calculate the probability of a given label (B, I, O) in sequence A given B. The model assigns a numerical weight to each feature, and then those weights are combined to determine the probability of A_k_. The conditional probability is calculated as follows:

3 $$ P\left( A\Big| B,\uplambda \right)=\frac{1}{Z(B)} exp\left({{\displaystyle {\sum}_k{\lambda}_k F}}_k\left(\mathrm{A},\ \mathrm{B}\right)\right) $$

where λ_k_ is a parameter to be estimated from training data and indicates the informativeness of the respective feature, Z(B) is a normalization factor and F_K_(A, B) = n i = 1 f_k_(A_i−1_, A_i_, B, i), where each f_k_(A_i−1_, A_i_, B, i) is either a state function s(A_i−1_, A_i_, B, i) or a transition function t(A_i−1_, A_i_, B, i).

#### Bidirectional CRF

In CRF labeling, we used the special forward and backward parsing techniques to dissect the entities in both directions. In forward parsing, the input tokens are read and tagged in their original order (left to right), while in backward parsing, it is done in the reverse order [18].
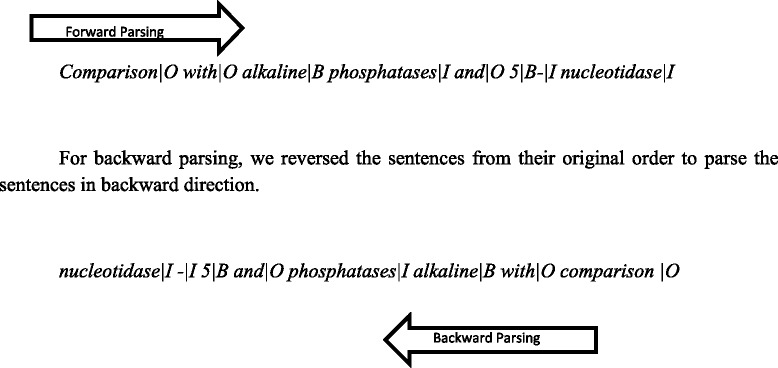


Further, we have used the second-order CRF for both forward and backward learning. In a higher order model of “n,” each label depends on a specific number of “n” previous labels. Thus, the probability will consider not only the previous observation and its features but also n-previous observations and features. Though the higher order models provide improved results, the training complexity of higher order models increases exponentially. So we used the second-order CRF which is generally used in NER task [2].

#### Model integration

The common ways to combine the results of bidirectional parsing includes simple set operations such as intersection and union. Usually, intersection will improve the precision and reduce the recall, while using union will improve the recall and reduce the precision [18]. In general, union and intersection methods failed to improve the performance because they lead to a trade-off between recall and precision. For better model integration, we used MIRA algorithm proposed by Crammer and Singer [19]. MIRA solves the above mentioned trade-off problem, since it combines the forward and backward models by adding the feature weights of both models [19]. Further, MIRA successfully reduces the training time by exploiting the no update procedure if the instance is classified as correct and also reduces the memory space by following no fixed step size for the update procedure. Hence, we have used MIRA for model combination and compared its performance with union in terms of both results and processing time.

MIRA is an online algorithm for multiclass classification problems. The objective of using MIRA is for building combined model with reduced training time and memory space. It is designed to learn a set of parameters (vector or matrix) by processing all the given training examples one-by-one and updating the parameters according to each training example, so that the current training example is classified correctly with a margin against incorrect classifications at least as large as their loss [20]. The change of the parameters is kept as small as possible.

4 $$ \begin{array}{c}\hfill \min \left\Vert {\omega}^{\left( i+1\right)}-{\omega}^{(i)}\right\Vert \hfill \\ {}\hfill score\left({x}_t,{y}_t\right) - score\left({x}_t,{y}^{\hbox{'}}\right)\ge L\left({y}_t,{y}^{\hbox{'}}\right)\forall {y}^{\hbox{'}}\hfill \end{array} $$

The score of the current correct training ***y*** must be greater than the score of any other possible *y*′ by at least the loss (number of errors) of that *y*′ in comparison to *y*.

### Post-processing

To further improve the performance of our system, we applied the following three post-processing techniques

1. Surrounding text features
2. Parenthesis mismatching
3. Abbreviation resolution algorithm

#### Surrounding text features

We used two different types of surrounding text features: (a) relation word features and (b) connective word features.

- *Relation words*: In biomedical text, existence of some relation keywords (binding, activate, etc.) implies that some protein names might occur [21]. We compiled around 400 interaction keywords from biomedical texts. If any relation keyword was present in the sentence, then its previous and next words were checked for protein/gene names occurring three or more times in the training set. They were then tagged as gene names if occurring so.
- *Connective words*: Similar to the relation word features, here, we checked for the linguistic cue connective words such as “and” and “or” in the sentence. If these words were present in the sentence, then the previous and next words were checked for protein/gene names that occur three or more times in the training set and tagged as gene names.

#### Parenthesis mismatching

One problem with CRF modeling is that it wrongly identifies the parenthesis, and it leads to parenthesis mismatching problem. For example, in the case of an opening curly brace being tagged and the closing curly brace not tagged, we need to remove the mismatched curly brace. We used left parenthesis and right parenthesis extension method to remove the mismatched parenthesis tagging [2]. This is shown in the following example 1.

Example 1:

*Sentence*: The hepatocyte nuclear factor-3 (HNF-3)/forkhead (fkh) proteins consist of an extensive family of tissue-specific and developmental gene regulators.

*Before post-processing*: (|B HNF|I -| I 3|I) |O

*After post-processing*: (|O HNF|B -| I 3|I) |O

#### Two-tier abbreviation algorithm

In abbreviation resolution, we used two popular techniques, namely (a) identification of the missed LF (long form) and SF (short form) [22] and (b) abbreviation disambiguation [23].

- Identifying the LF (long form) and SF (short form): CRF tagger most of the time tags only long form or short form and misses either one. To tackle this and identify the missed long-form and short-form abbreviations, we used a modified version of the simple abbreviation algorithm which is used in BioC [22] and named as “extract abbreviation method”. This is shown in the following Example 2.
*Example 2:*
*Sentence:* Brown adipose tissue (BAT) and brown-like cells in white adipose tissue (WAT) can dissipate energy.
*Before Post-processing:* Brown |O adipose |O tissue |O (|B BAT|I) |I
*After Post-processing:* Brown |B adipose |I tissue |I (|B BAT|I) |I
- Abbreviation disambiguation: The second technique in two-tier abbreviation algorithm is abbreviation disambiguation. Sometimes two proteins or two genes have the same abbreviation, for example, “*angiotensin converting enzyme (ACE)*”and “*acetylcholinesterase (ACE)*.”*.*In the above example, “ACE” denotes both angiotensin converting enzyme (ACE) and acetylchlinesterase (ACE). To overcome this problem, we used the abbreviation disambiguation method word sense disambiguation (WSD) [23]. In WSD, along with other features, we used domain specific features such as CUI (concept unique identifiers) [24] and MeSH terms [25]. WSD identifies all related words in the text which could be mapped to CUI or MeSH terms and disambiguates them to their correct sense of the long form or short form.

## Results and discussions

BCC-NER training and testing is based on BioCreative II GM corpus which contains 15,000 training sentences and 5000 testing sentences [7]. While training and testing, we employed our feature set with bidirectional CRF models in both forward and backward directions. Finally, we applied MIRA algorithm to integrate both models to construct the combined model.

The measures of precision, recall and F-scores were used to evaluate the performance of learning models. The following four different learning were performed

1. CRF + Forward parsing + post-processing,
2. CRF + Backward parsing + post-processing,
3. CRF+ Union (Forward + Backward) + post-processing and
4. CRF + Combined model MIRA + post-processing.

We carried out our experiment with the following performance measure criteria using the three equations given below.

5 $$ P r e c i s i o n( P)= T P/\left( TP+ FP\right) $$

6 $$ Recall(R)= T P/\left( TP+ FN\right) $$

7 $$ \mathrm{F}\hbox{-} \mathrm{score}=2\times P\times R/\left( P+ R\right) $$

Where, TP refers to the number of proportion of biological entities correctly identified by our hybrid approach, FN refers to the number of proportion of biological entities that the approach failed to identify and FP refers to the number of proportion of biological entities that were incorrectly identified by this approach

Table 2 represents the results of the evaluation in BioCreative II GM test corpus. The results illustrate that our hybrid approach accomplishes improved results. In forward parsing model, it gives an F-score of 86.21 and in bidirectional combined model using union method it improves the F-score to 86.51. The final combined model with MIRA algorithm resulted in a higher F-score of 86.95. The additional advantage of using MIRA is in terms of training time. The training time of both forward and backward model in a Linux sever with 48 GB RAM was approximately 4 h. The training time of the combined model using union model was about 7 h. However, the MIRA combined model completed the training in 5 h.

**Table 2:** System performance on various models

Learning model	Precision	Recall	F-measure
CRF + forward parsing + post-processing	89.18	83.45	86.21
CRF + backward parsing + post-processing	89.38	83.55	86.36
CRF+ union (Forward + backward) + post-processing	89.58	83.65	86.51
CRF + combined model MIRA + post-processing	89.95	84.15	86.95

Comparison with other systems:

Table 3 shows the performance comparison of our system with other open source machine learning systems in BioCreative II GM corpus [26]. While LingPipe, ABNER and BANNER systems uses single CRF model with different feature set for gene/protein mentions. Our system attains better results by using combined MIRA model with rich set of features and post-processing module. The unique features and methodology of our system which contributes performance improvement is discussed in the following section.

**Table 3:** Comparison of our system with other open source systems

System	Precision	Recall	F-measure
LingPipe	72.95	88.49	79.97
ABNER	86.93	51.49	64.88
BANNER	88.66	84.32	86.43
BCC-NER	89.95	84.15	86.95

## Discussions

In this paper, we describe our hybrid named entity recognition system named BCC-NER for tagging biomedical entities. BCC-NER integrated all major happenings in current NER task and includes three modules. For example, we have used a rich set of features combining the major 32 basic ones, word N-grams, and corpus frequency words. The state-of-the-art feature selection and extraction algorithm PCA was applied to reduce the high number of features associated with each tokens.

The latest results on biomedical NER clearly indicate that better performance can be achieved by combining several systems. In these lines, BCC-NER employs bidirectional CRF model combined with MIRA. We are the first one to explore such a combination using MIRA in biomedical NER which gives improvised outcomes than the traditional union and intersection methods. Another important feature that contributes to our hybrid approach is the consideration of contextual clues. In contextual clues, our tagger finds the interaction words and checks if the previous and next words are present in the post-keyword list (pkl). If present, it is tagged as a gene.

For example,

*Before post-processing*:

*Early complement components*, *<gene>C1q</gene> and C4*, *and IgA secretory piece were absent.*

In the above example, our contextual clues find the connective word “and,” and then checks for the previous and next words for their presence in pkl. The previous word C1q has been already tagged as gene. So the next word “c4” is then checked for its presence in pkl and tagged as gene after this step.

*After post-processing*:

*Early complement components*, *<gene>C1q</gene> and <gene>C4</gene>*, *and IgA secretory piece were absent.*

Following this step, we re-implemented the same step again but with relation words. If any relation keywords are found in the sentence, then both its previous and next words were checked for protein/gene names occurring three or more times in the training set.

Finally, we applied the parenthesis post-processing and two-tier abbreviation algorithm as explained above in the Sections 2.3.2 and 2.3.3. Thus, the issues of parenthesis mismatching and abbreviation disambiguation were subdued.

## Conclusions

We propose a hybrid named entity tagging approach BCC-NER which on evaluation indicates that the inclusion of rich set of features and utilization of bidirectional CRF combined with MIRA gives best results. Additional performance improvement was achieved by post-processing steps including surrounding text features, parenthesis mismatching and two-tier abbreviation algorithm.

Although in BCC-NER we tried to integrate various state-of-the-art methods on existing tools, some aspects can be further explored. We are currently investigating other approaches including domain knowledge information through the use of dictionaries or machine learning-based solutions. In addition, we plan to include methods like co-training, superior abbreviation algorithms and different features to generate improved results.

## References

[1] Settles B (2005). ABNER: an open source tool for automatically tagging genes, proteins and other entity names in text. Bioinformatics.

[2] Leaman R, Gonzalez G (2008). Pacific symposium on biocomputing. BANNER: an executable survey of advances in biomedical named entity recognition.

[3] Campos D, Matos S, Oliveira JL (2013). Gimli: open source and high-performance biomedical name recognition. BMC Bioinformatics.

[4] Raja K, Subramani S, Natarajan J (2014). A hybrid named entity tagger for tagging human proteins/genes. Int. J. Data Min. Bioinform..

[5] Zhu F, Shen B, Combined SVM-CRFs for Biological Named Entity Recognition with Maximal Bidirectional Squeezing. PLoS ONE. **7**(6) (2012) 10.1371/journal.pone.0039230 PMC3383748 22745720

[6] Chang JT, Schutze H, Altman RB (2004). GAPSCORE: finding gene and protein names one word at a time. Bioinformatics.

[7] Tanabe L, Xie N, Thom LH, Matten W, Wilbur WJ, GENETAG: a tagged corpus for gene/protein named entity recognition. BMC Bioinf. **6**(1) (2005). 10.1186/1471-2105-6-S1-S3 PMC1869017 15960837

[8] Atserias J, Casas B, Comelles E, González M, Padró L, Padró M (2006). Proceedings of LREC. FreeLing 1.3: Syntactic and semantic services in an open-source NLP library.

[9] Liu H, Christiansen T, Baumgartner WA, Verspoor K (2012). BioLemmatizer: a lemmatization tool for morphological processing of biomedical text. J Biomed Semant.

[10] Jiampojamarn S, Cercone N, Kešelj V (2005). Conf. of the Pacific Assoc. for Computational Linguistics. Biological named entity recognition using n-grams and classification methods.

[11] Moe RE (2014). International Conference on Text, Speech, and Dialogue. Clustering in a News Corpus.

[12] Lu Y, Cohen I, Zhou XS, Tian Q (2007). Proceedings of the 15th ACM International Conference on Multimedia. Feature selection using principal feature analysis.

[13] Bu LH, Li GZ, Zeng XQ (2007). Reducing error of tumor classification by using dimension reduction with feature selection. Lecture Notes Operations Res.

[14] Saha SK, Mitra P, Sarkar S (2012). A comparative study on feature reduction approaches in Hindi and Bengali named entity recognition. Knowl.-Based Syst..

[15] Bengio Y, Courville A, Vincent P (2013). Representation learning: a review and new perspectives. IEEE Trans. Pattern Anal. Mach. Intell..

[16] Lafferty J, McCallum A, Pereira F (2001). Proceedings of the Eighteenth International Conference on Machine Learning. Conditional random fields: Probabilistic models for segmenting and labeling sequence data.

[17] Klinger R, Friedrich CM (2009). Proceedings of RANLP. Feature Subset Selection in Conditional Random Fields for Named Entity Recognition.

[18] Hsu C-N, Chang Y-M, Kuo C-J, Lin Y-S, Huang H-S, Chung I-F (2008). Integrating high dimensional bi-directional parsing models for gene mention tagging. Bioinformatics.

[19] Crammer K, Singer Y, Ultraconservative online algorithms for multiclass problems. J. Mach. Learn. Res. **3**, 951–991 (2003)

[20] McDonald R, Crammer K, Pereira F (2005). Proceedings of the 43rd Annual Meeting on Association for Computational Linguistics. Online large-margin training of dependency parsers.

[21] Yang Z, Lin H, Li Y (2008). Exploiting the contextual cues for bio-entity name recognition in biomedical literature. J. Biomed. Inform..

[22] Comeau DC, Dogan RI, Ciccarese P, Cohen KB, Krallinger M, Leitner F, Lu Z, Peng Y, Rinaldi F, Torii M, Valencia A, Verspoor K, Wiegers TC, Wu CH, Wilbur WJ, BioC: a minimalist approach to interoperability for biomedical text processing. Database. (2013) bat064 10.1093/database/bat064 PMC3889917 24048470

[23] Stevenson M, Guo Y, Al Amri A, Gaizauskas R (2009). Proceedings of the Workshop on Current Trends in Biomedical Natural Language Processing. Disambiguation of biomedical abbreviations.

[24] McInnes BT, Pedersen T, Carlis J (2007). AMIA Annual Symposium Proceedings. Using UMLS Concept Unique Identifiers (CUIs) for word sense disambiguation in the biomedical domain.

[25] Nelson, S. J., Powell, T., Srinivasan, S., Humphreys, B. L, Unified Medical Language System (UMLS) Project. In Encyclopedia of library and information sciences pp. 5320–5327(2010).

[26] Wang, X., Yang, C., Guan, R., A comparative study for biomedical named entity recognition. Int. J. Mach. Learn. Cybern., 1–10 (2015) doi:10.1007/s13042-015-0426-6

